# Cost-Effective Trap qPCR Approach to Evaluate Telomerase Activity: an Important Tool for Aging, Cancer, and Chronic Disease Research

**DOI:** 10.6061/clinics/2021/e2432

**Published:** 2021-02-01

**Authors:** Thalyta Nery Carvalho Pinto, Juliana Ruiz Fernandes, Liã Barbara Arruda, Alberto José da Silva Duarte, Gil Benard

**Affiliations:** I Laboratorio de Dermatologia e Imunodeficiencias (LIM56), Faculdade de Medicina FMUSP, Universidade de Sao Paulo, Sao Paulo, SP, BR; II Division of Infection and Immunity, Center for Clinical Microbiology, Royal Free Hospital Campus, London, University College London, Division of Infection and Immunity, Center for Clinical Microbiology, Royal Free Hospital Campus, LondonUniversity College London UK; III Laboratorio de Micologia Medica, Instituto de Medicina Tropical, Universidade de Sao Paulo, Sao Paulo, SP, BR

**Keywords:** Telomerase, Telomere, Aging, Real-Time Polymerase Chain Reaction

## Abstract

**OBJECTIVES:**

Telomeres are a terminal “DNA cap” that prevent chromosomal fusion and degradation. However, aging is inherent to life, and so is the loss of terminal sequences. Telomerase is a specialized reverse transcriptase encoded by self-splicing introns that counteract chromosome erosion. Telomerase activity is observed during early embryonic development, but after the blastocyst stage, the expression of telomerase reduces. The consequences of either insufficient or unrestrained telomerase activity underscore the importance of ongoing studies aimed at elucidating the regulation of telomerase activity in humans. In the present study, we aimed to standardize a simplified telomerase repeat-amplification protocol (TRAP) assay to detect telomerase activity in unstimulated and PHA-stimulated mononuclear cells.

**METHODS and RESULTS:**

Our optimized qPCR-based can efficiently evaluate telomerase activity. Quantification of protein and DNA between unstimulated and PHA-stimulated peripheral blood mononuclear cells revealed cellular activation and cell-cycle entry. The assay also showed that relative telomerase activity is significantly different between these two conditions, supporting the applicability of the assay. Furthermore, our findings corroborated that telomerase activity decreases with age.

**CONCLUSIONS:**

Telomeres and telomerase are implicated in aging and development of chronic diseases and cancer; however, difficulty in accessing commercial kits to investigate these aspects is a critical constraint in health surveillance studies. Our optimized assay was successfully used to differentiate telomerase activity between unstimulated and stimulated cells, clearly showing the reactivation of telomerase upon cell activation. This assay is affordable, reproducible, and can be executed in resource-limited settings.

## INTRODUCTION

Telomeres are chromosome ends that function as an “end protection” against fusions and degradation. Telomeric repeats consist of a guanosine-rich strand and a cytosine-rich strand. The loss of these terminal sequences is inherent to life. There is a replication problem with the impossibility of full reproduction of linear DNA ends, which can be attributed to certain peculiarities of the structure and function of the DNA replication machinery. Evolution has designed a mechanism to counteract telomere shortening, *i.e.*, specialized reverse transcriptase encoded by self-splicing introns known as telomerase. Telomerase is a ribonucleoprotein complex, and the template for telomeric repeat synthesis is localized within the integral RNA subunit (1-6).

Telomerase activity is variable while also being tightly regulated. Telomerase activity is present during early embryonic development, but after the blastocyst stage, the expression of telomerase decreases and cannot be detected in neonatal somatic cells. Human embryonic stem cells have the most robust telomerase activity, which means that these cells expand their proliferative capacity (7). In somatic cells, telomerase activity can be detected during proliferation, but this activity is inadequate for preventing telomere shortening. Insufficient telomerase activity has also been associated with certain diseases, such as bone marrow failure syndrome and pulmonary fibrosis, while aberrant reactivation of telomerase activity is associated with particular diseases, such as cancer (8-11).

In mature somatic cells, *i.e.*, blood cells, telomerase activity is very low or non-existent. In hematopoietic cells and cells with stemness properties, *i.e.*, most stem cells and tumor cells, enzyme activity is high. However, telomerase activity *per se* is insufficient to maintain telomere length during aging (12). Interestingly, telomerase activity has been inversely correlated with telomere length, and short telomeres are associated with high telomerase activity. Short telomeres in many human cells can trigger telomerase activity and promote telomere extension, while cells that start to lose their telomere repeats without telomerase compensation enter into replicative senescence (13). In addition, telomerase also plays an important role in aging, thus telomerase has functions other than telomere maintenance (14-16).

Studies aimed at identifying a correlation between constitutive telomerase activity in peripheral blood mononuclear cells (PBMCs) and antiproliferative therapies, specific diseases, and age identified very low expression of telomerase. In fact, resting PBMCs also express low levels of telomerase, and it is possible that actual expression would be missed for being below the detection limit of the assay. In addition, telomerase expression varies as a function of the cell-cycle stage, exposure to inflammatory mediators, stress hormones or oxidative stress (17-21). Telomerase activity increases in antigen-or mitogen-stimulated cells. Thus, a stimulated condition is a way to overcome the limitation associated with the unstimulated condition, *i.e.*, being able to indicate individual differences in the overall capacity of immune cells to respond to a relevant challenge (22,23).

Upon activation, lymphocytes exhibit enhanced telomerase activity. Crosslinking TCR and CD28 with either cognate antigens, lectins, or antibodies triggers a signaling cascade that induces the translocation of transcription factors (*e.g.*, NF-κB) into the nucleus, initiating IL-2 production, and stimulating cell-cycle entry and DNA synthesis (24,25). Increased telomerase activity also results in triggering the immune responses and prevents immune cell senescence during clonal-cell expansion (26).

Assays for the detection of telomerase activity can employ two strategies. Less commonly, telomerase activity can be measured based on amplification of telomerase signals, which involves quantification using either enzyme-linked immunosorbent assay (ELISA), electroluminescence or a fluorometric optosensor. These types of assays are time-consuming, less sensitive, and require specific equipment for reading results, for example a fluorescence microscope or fluorimeter (7). The most commonly employed strategy is based on the detection of telomerase amplicons through PCR-based assays. A specific PCR-based assay known as the telomerase repeat-amplification protocol (TRAP) was developed to determine the expression of telomerase in cell lines and cancer biopsy samples, but this assay was not optimized for normal cells that have low telomerase expression.

Morin (27) measured telomerase activity using a direct telomerase activity assay; this strategy involved subjecting HeLa extract to cycles of heating, followed by another round of extraction and electrophoresis. The results were visualized over a period of two to four days by autoradiography. Counter et al. (28) followed the Morin (27) protocol but instead used human embryonic kidney (HEK) cells and evaluated the results of electrophoresis through the measurement of mean population doublings.

Kin and Wu (29) used the TRAP assay to evaluate tumor extracts by protein extraction with CHAPS buffer, followed by a thermocycler stage and electrophoresis. Wege et al. (30) compared the use of *in-house* real-time quantitative TRAP (RQ-TRAP) and commercially available kits using different cell lines and obtained a significant positive correlation between the methods. RQ-TRAP assays in general target endogenous telomerase by adding a number of telomeric repeats at the 3’ end of a substrate oligonucleotide. The products extended by telomerase are then amplified by PCR and serve as a readout of telomerase activity (31).

Hiyama et al. (32) demonstrated that neuroblastomas characterized by samples lacking telomerase activity had a favorable clinical prognosis, suggesting that telomerase activity could be employed as a prognostic marker. More recently, telomerase activity has been determined in a range of cancer types (33,34) and has been utilized as a robust biomarker for therapy and tumor progression. The commercial kit is not recommended for the evaluation of telomerase activity in tissues, precluding its use in tumor biopsies. In contrast, *in-house* TRAP assays can be used to evaluate telomerase activity in tissues and tumor biopsy samples (35). *In-house* qPCR also outperforms commercial PCR-ELISA kits in terms of efficacy because PCR-based protocols are more sensitive and do not require complex multi-step procedures. Importantly, evaluation of telomerase expression using qPCR involves threshold cycle, a highly sensitive quantitative measurement. The results are obtained during the exponential phase and as such are not affected by reaction compounds, which in certain conditions can be limiting (31).

The consequences of either insufficient or unrestrained telomerase activity underscore the importance of ongoing studies aimed at determining the regulation of human telomerase activity. Telomeres and telomerases are implicated in aging and development of chronic diseases and cancer as they are involved in important processes; they could also be used as markers of senescence. The difficulty in accessing commercial kits to evaluate telomerase activity, a critical constraint for health surveillance studies, *i.e.*, it is interesting to use an existing *in-house* assay. This could be explored as a relevant tool for assessing the of aging. Therefore, in the present study, we aimed to standardize a simplified TRAP assay to detect telomerase activity in unstimulated and PH A-stimulated mononuclear cells.

## MATERIAL AND METHODS

### Volunteers

Adults (18-75 years old), without chronic diseases related to the immune system, were enrolled in the study after obtaining informed consent. The study was approved by the School of Medicine of the University of São Paulo Ethics Committee (#1956268) and was performed in accordance with the Helsinki Declaration. A total of 20 mL of peripheral blood from each volunteer was collected in heparinized tubes.

### Culturing of Peripheral Blood Mononuclear (PBMCs) and HEK Cells

PBMCs were isolated using the Ficoll Hypaque (GE Healthcare Life Sciences, Boston, United Kingdom) density gradient and stored in liquid nitrogen. PBMCs were thawed and maintained in culture flasks containing RPMI (GIBCO, Massachusetts, United States) and 10% AB* serum (Sigma-Aldrich, Steinhein, Germany) for 24h. Subsequently, PBMCs were cultured in a 24-well plate under unstimulated conditions (medium only), and PHA-stimulated (25 μg/mL, Sigma-Aldrich, Steinhein, Germany) conditions for three days. Afterwards, 2,000,000 cells were collected in nuclease-free 1.5 mL-microfuge tubes. Cells were washed with PBS without calcium or magnesium and pelleted by centrifugation at 8000 x *g* for 5 min at room temperature (37°C). The supernatant was carefully discarded to ensure that all residual liquid was removed, and the pellet was used for cell lysis.

**Figure 1:**
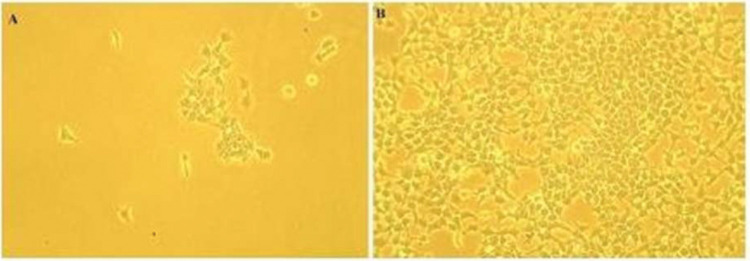
HEK Cell Culture. **A:** HEK cell cultures showing the start of culture with a low cell density. **B:** Cells after seven days of culture showing 85% confluence.

HEK 293 cells were used as positive controls as this cell line overexpresses telomerase (36). HEK cells were cultured in culture flasks (25 and 75 cm^3^) using DMEM (GIBCO, Massachusetts, United States) supplemented with 10% fetal bovine serum (FBS) (GIBCO, Massachusetts, United States) until they became 85% confluent (Figure 1). Subsequently, the cells were split into two culture flasks for further growth until a count of 1x10^8^ cells was reached. HEK cells were then pelleted following the same procedure described for the PBMCs.

### Cell Lysis Procedure and Protein Quantification

CHAPS or NP-40 lysis buffers could be used during this procedure. While a CHAPS buffer is described as being important for maintaining protein activity, NP-40 renders the extraction step more efficient. For the present purpose, we used a CHAPS buffer because measurement of telomerase activity was more important than that of its yield. Hence, each of the 2,000,000 pellets of PBMCs and HEK cells were resuspended in 50 μL ice-cold CHAPS lysis buffer (Cell Signaling, Danvers, United States) containing RNase inhibitor (40 U/μL) and protease inhibitor cocktail (1×) (Thermo Fisher, Massachusetts, United States), and then stored at -80°C followed by two freeze-thaw cycles to promote cell lysis. After the second thaw cycle, cells were centrifuged at 16,000 × *g* for 10 min at 4°C. Approximately 90% of the supernatant volume was collected in a fresh nuclease-free microtube tube, ensuring that no traces of cell debris from the pellet were present.

Protein and DNA concentrations in PBMC and HEK lysates were quantified using NanoDrop ND-100 (Uniscience, Osasco, Brazil) immediately after cell lysis and then the samples were frozen at -80°C. The A280 protocol from NanoDrop ND-100 (Uniscience, Osasco, Brazil) was used for protein quantification. DNA concentration was measured using the DNA protocol provided along with the equipment and sample concentration was determined as ng/μL based on absorbance readings at 260 nm and the selected analysis constant.

### qPCR

Previously, methods have been proposed to measure telomerase expression based on primer extension; however, these methodologies were fraught with low sensitivity, especially in cells with decreased expression of telomerase (37). The TRAP protocol was based on PCR and described in 1997 by Kim et al. (29). Moreover, this protocol is based on the exponential amplification of primer-telomeric repetitions that the telomerase generates during PCR. The enzyme lengthens an oligonucleotide model (TS primer) to form telomeric-lengthening products. To maximize detection, these products were amplified by PCR. One limitation of PCR is the necessity of a minimum template length for the reverse primer to hybridize and start the reaction efficiently. Only products that have been elongated by four or many telomeric repetitions are detectable (38-40).

To ensure the efficiency of the assay, HEK lysate was employed as a high telomerase activity control. At least five separate standard curves were assessed using 1:10 serial dilutions from one million HEK lysates. Additionally, lysis buffer and ultrapure water exclusively were used as negative controls to verify the presence of contamination.

PCR was performed using Luna SYBRGreen 1× (New England Biolabs, Ipswich, USA) and 0.25 μM of each primer: TS (5′-AATCCGTCGAGCAGAGTT-3′) and ACX (5′-GCGCGG(CTTACC)3CTAACC-3′). (29,37) in a final reaction volume of 25 μL. Cell lysates (0.15 mg/mL protein) were used as the template (n=32), while for the standard curve (HEK), the number of cells was used as input (starting at 10^6^ cells). PCR was performed on an Applied Biosystems 7500 Real-Time PCR system (Applied Biosystems, Waltham, USA). The cycle conditions were as follows: one cycle at 95°C for 60 s followed by 40 cycles of 15s at 95°C, 30s at 60°C, and an additional melting curve cycle.

### Statistical Analysis

Statistical analysis was conducted using GraphPad Prism 5.0 (GraphPad Software Inc., San Diego, USA). The distribution of data (parametric or non-parametric) for each parameter was tested using the KS test. To assess parametric data, we used the unpaired *t*-test with Welch’s correction, while unstimulated and stimulated conditions were assessed using a paired *t*-test. For non-parametric data, we used the Mann-Whitney test. Graphical representation shows medians with interquartile ranges. Differences were considered significant when the *p-value* was <0.05.

## RESULTS

PBMCs from 32 volunteers (mean age: 45 years; range: 19-71, SD ±22) were evaluated. After 72h of culture in the presence of medium only or PHA, PBMCs were lysed, and protein and DNA quantification was established using NanoDrop ND-100 (Uniscience, Osasco, Brazil). DNA and protein concentrations in the samples are shown in Table 1. PHA-stimulated cells exhibited significantly higher concentrations of both DNA (134.7±24.0 ng/mL, *p*=0.0002) and protein (45.5±5.0 mg/mL, *p*<0.0001) compared to unstimulated (577.0±104.2 ng/mL and 26.4±6.8 mg/mL, respectively) as a result of activated cell proliferation and increased protein synthesis.

**Figure 2:**
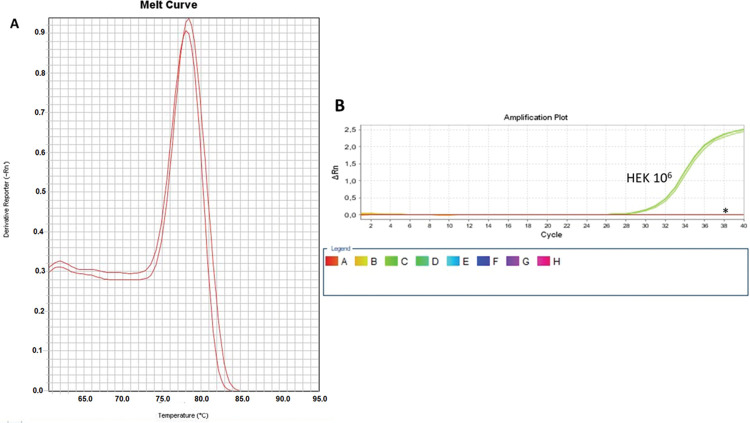
Amplification and Specificity Test of HEK Cell Line Lysate. qPCR assay to assess telomerase activity in HEK cell lysates. A representative plot of 10^6^ cell lysate amplification. **A**: Melting curve of qPCR run showing one product of amplification. **B**: Amplification of the 106-cell lysate plot showing a well-defined exponential linearity and a plateau demonstrating that the sample reached the limit of the equipment warranting sample positivity. Negative controls were represented by CHAPS lysis buffer and ultrapure water (*).

**Figure 3:**
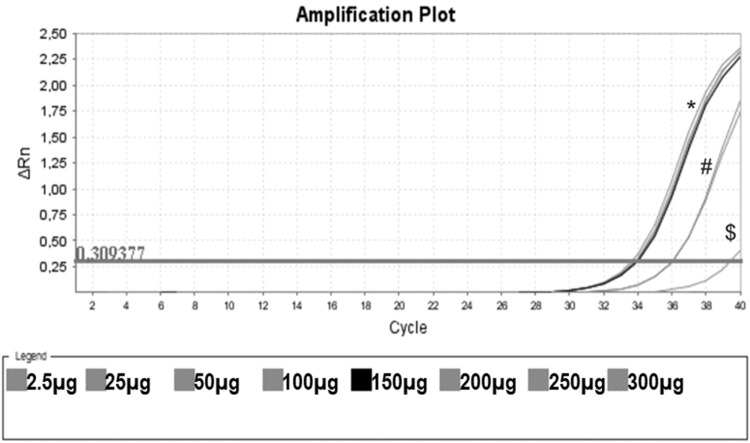
Protein Titration with One Unstimulated Sample. Protein titration of one unstimulated volunteer sample, initiating on the protein concentration of 0.0025 mg/mL (no amplification), 0.025 mg/mL ($), 0.050 mg/mL, 0.100 mg/mL (#), 0.150 mg/mL (black line) and 0.20 mg/mL, 0.25 mg/mL and 0.30 mg/mL (*). In this assay, CHAPS and ultrapure water were also used as negative controls (no amplification).

As a control, we used the protein lysate obtained from 10^6^ HEK lineage cells as described by Hou et al. (37). HEK cells have the advantage of providing higher yields of protein (on average 8.340 mg/mL) compared with PBMC samples (Table 1). Amplification specificity was confirmed by assessing the melting curve (Figure 2). The melting curve was used to determine whether the intercalating dye assays produced a single and specific product. Amplification specificity is a serious concern with intercalating dye assays because they can bind to any double-stranded DNA product, including nonspecific amplicons. The HEK sample featured a well-defined exponential amplification, showing no nonspecific peaks that could represent contamination or unspecific binding (Figure 2B). Only on the standard curve (HEK), the template input was based on the absolute number of cells used for lysate rather than the protein concentration. For the volunteers, the same protein input (0.15 mg/mL) was employed for all tested samples. Increasing the input of HEK lineage to 10^7^ cells did not result in an earlier start of the exponential amplification (not shown); instead, reaction saturation was observed. An important issue during the qPCR assay was optimizing the appropriate template input for the best amplification. Protein template input was highly variable in the published protocols, and in some publications, only the number of lysed cells was provided, especially when commercial kits were used (37,41-47). We performed a protein titration assay, starting from a very low concentration (0.0025 mg/mL) (Figure 3). The protein concentration of the samples of the volunteers was inferred using NanoDrop, and then diluted in ultrapure nuclease-free water from 0.0025 mg/mL to 0.3 mg/mL. The objective of this step was to find an optimal protein concentration to be used in all experiments to ensure that each well of each tested sample would have the same input of protein (template) across all assays.

Figure 3 shows that low protein input (0.0025 mg/mL) was insufficient to elicit amplification. By increasing the input to 0.025 and 0.050 mg/mL, we obtained some amplification, but the curve did not reach a plateau. When the protein concentration tested was raised from 0.15 mg/mL to 0.30 mg/mL, a clear amplification plot (exponential, linear, and the beginning of the plateau) was observed. Based on these findings, we decided to use 0.15 mg/mL as protein input for subsequent assays.

**Figure 4:**
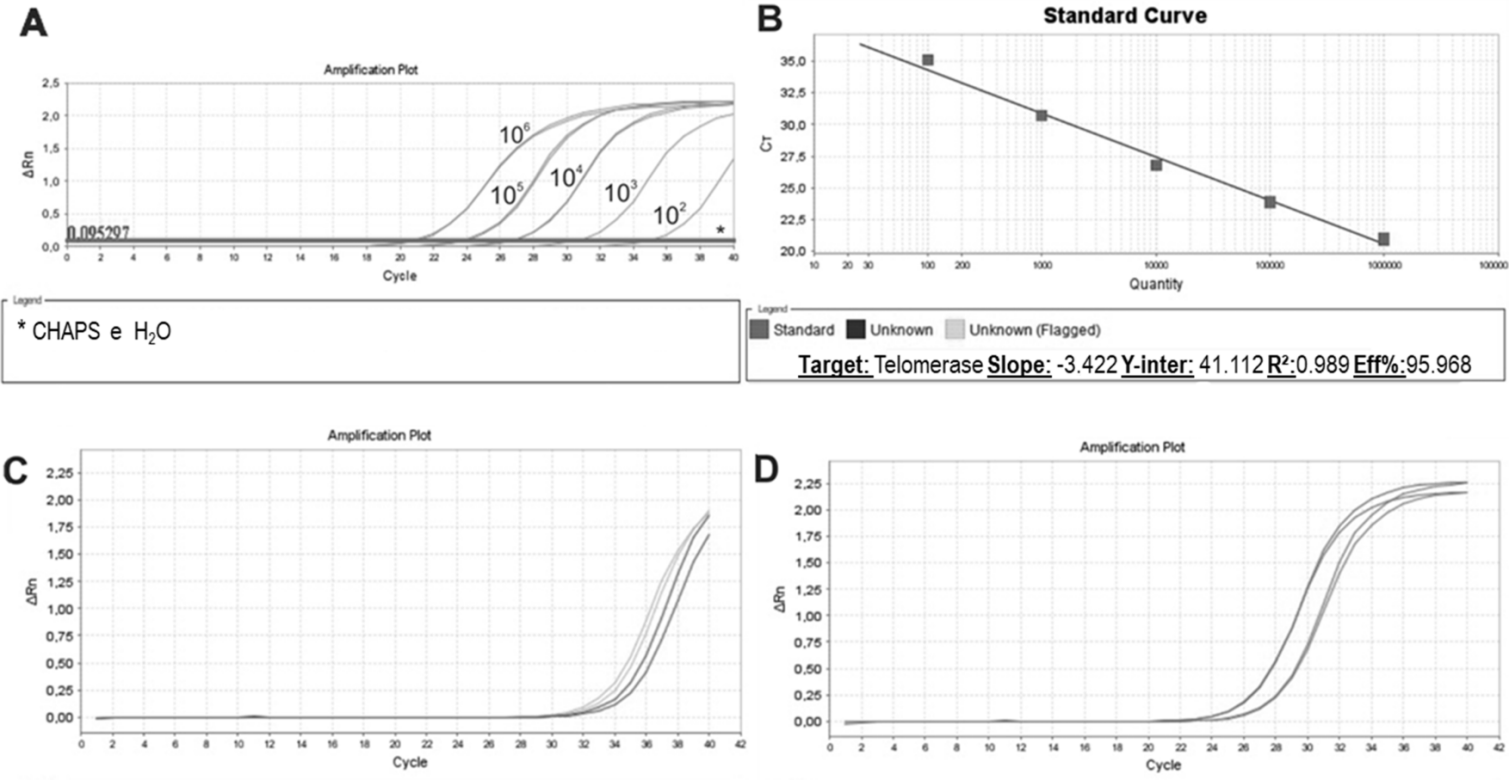
Standard Curve, Unstimulated and PHA-stimulated Sample Amplification. qPCR run of standard curve and two samples from healthy individuals. **A**: Ten- fold serial dilution of at least five template concentrations of HEK cells using CHAPS buffer and ultrapure water as negative controls (*). **B**: Standard curve parameters showing an efficiency of 95.968%, slope of -3.42 and r^2^ of 0.989. **C**: Unstimulated sample amplification. **D**: PHA-stimulated sample amplification.

We then established a standard curve (Figure 4A and 4B). The standard curve was based on the number of HEK cells using a 10-fold serial dilution series of at least five dilution points, ranging from 10^2^ to 10^6^ lysed cells. Each reaction was performed in triplicate. The HEK cell standard curve allowed us to quantify the relative activity of telomerase enzyme because this cell line exhibits higher signal than other cell lines for overexpressing telomerase (36).

The standard curves showed an assay efficiency of 95.9% with an r^2^ of 0.989 and slope of -3.4, complying with the gold standard parameters recommended for the relative quantification of samples: assay efficiency between 90% and 110% and an r^2^ between 0.98 and 1. The r^2^ indicates the accuracy of the prediction of a value on x based on the value of y. r^2^ values >0.98 indicate strong confidence in correlating two values. An ideal slope is -3.32, but slopes between -3.1 and 3.6 are acceptable and consistent with efficiencies between 90% and 110%. Slopes different from this range can indicate problems in sample quality or pipetting.

Once the standard curve was established, unstimulated and PHA-stimulated PBMC samples were evaluated. As expected for resting cells, unstimulated samples (Figure 4C) exhibited a delayed amplification compared to PHA-stimulated samples (Figure 4D), showing a low quantity of telomerase that entered the cell cycle, actively proliferating, and induced telomerase activity.

The final result was expressed as relative telomerase activity (RTA), the percentage of telomerase activity based on the standard curve sample (HEK cells) (37,44,46). The Y-intercept and slope values from each PCR equation were used to quantify the RTA of the unknown samples (=10[(Ct sampleYint)/slope]). To ensure correct inferences, a standard curve was generated for each assay.

**Figure 5:**
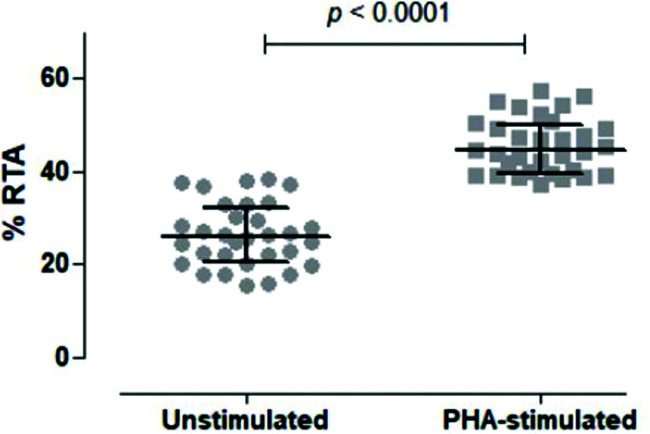
Comparison of Unstimulated and PHA-stimulated Relative Telomerase Activity (RTA). Comparison of unstimulated and PHA-stimulated conditions in 32 healthy subjects using paired t-test. The mean %RTA was 26.35% for unstimulated and 45.53% for PHA-stimulated cells. The confidence interval of 95% was not shared by unstimulated (23.89-28.82) and PHA-stimulated (43.38-47.68) conditions, confirming that the data were significantly different and not overlapping.

We then tested unstimulated and PHA-stimulated PBMCs from 32 volunteers to evaluate whether the assay was sensitive and specific enough to differentiate between the two conditions. As shown in Figure 5, the assay clearly quantified telomerase activity in resting and activated mononuclear cells, respectively, with a significant difference between the two conditions (*p<*0.0001).

As aging is one of the factors influencing telomere shortening, we tested whether there was a correlation between telomerase activity and donor age. PHA-induced increase in telomerase activity measured *per se* (*r*=-0.51; *p*=0.003) or delta (telomerase activity of unstimulated cells subtracted) showed a discrete inverse correlation with age (*r*=-0.52; *p*=0.002).

## DISCUSSION

We optimized a qPCR assay, based on a standard curve of an immortalized cell line, capable of distinguishing telomerase activity from activated and resting PBMCs. Evaluation of telomerase activity can provide relevant clinical information as alterations of telomerase activity and have been implicated in many physiological and pathological processes underlying aging, cancer and chronic illnesses.

Measurement of telomerase activity is not trivial. Getliffe et al. (48) showed that telomerase measurements can be severely affected by experimental variation as telomerase is a single-stranded RNA enzyme that can readily lose its activity upon handling. Experimental procedures for telomerase activity assessment require careful manipulation to avoid contamination with enzyme inhibitors, foreign DNA/RNA and external proteins. Telomerase activity expression can vary between samples, especially regarding variability in the metabolic state of the cell (*e.g.*, resting *vs.* activated). Our findings with PHA-stimulated and unstimulated cells illustrate this variation, corroborating the findings of Yamada et al. (49), but this time with a higher number of individuals.

We used the HEK cell line to generate the standard curve of the assay, as described in previous studies, because it exhibits high and stable telomerase activity as well as quick and easy expansion and maintenance. The selection of the control cell line must be considered carefully because there are certain immortal telomerase-negative cell lines that exhibit great variability in telomere lengths that are not under the control of telomerase (50). Telomerase reactivation is a critical limiting step in cancer development and in immortalization cell processes that maintain or upregulate the function of the enzyme (35). Thus, the choice of cell lineage and experimental controls takes into account this reactivation, which contributed to our choice of the immortal cell line, HEK, for designing a standard curve.

Deregulation of telomerase function has already been described in some diseases, resulting in an accelerated increase in telomere length and proliferative deficiencies. Defects in the enzyme can generate genetic instability and increase the incidence of cancer. As such, increased expression and activity of telomerase can confer an unlimited replicative potential, a feature of malignant cells, but not in somatic cells. Inhibition, on the other hand, can lead to telomere shortening and apoptosis (51). Therefore, this type of assay can be used as an important tool for research on cancer topics.

In normal somatic cells, telomerase activity is tightly regulated during development; enzyme activity is lost during embryological differentiation, with the exception of a few cell types, such as germinative cells, activated lymphocytes and stem cells that can maintain telomerase activity (15). The presence of telomerase in hematopoietic cells is related to the proliferative status of the cells (43). When the quiescent state and further activated lymphocytes were compared, telomerase activity increased in the activated state (11). One of the first attempts to measure telomerase activity in PBMCs employed both unstimulated and PHA-stimulated leukocytes, and a PCR. Telomerase activity was not detected in unstimulated cells, but upon PHA stimulation, low levels of enzyme activity were detected at 24h, while a 10-fold increase was seen after 48h (24). Yamada et al. (49) also evaluated stimuli other than PHA and showed that lymphocytes, but not polymorphonuclear leukocytes and monocytes, enhance telomerase activity after activation.

The importance of telomerase upregulation is reinforced by recent studies showing that disruption of telomerase activity in unstimulated PBMCs from healthy donors results in cell proliferation, restricted lifespan, and altered telomere structure (‘telomere uncapping’) without changing the rate of overall telomere shortening (52). The proliferative potential of cells is connected directly with telomere and telomerase dynamics as well as with cellular aging as both telomere and telomerase activity are limited. Besides, disease mechanisms commonly involved in the aging process can be implicated at both the molecular and cellular levels of telomere and telomerase dynamics (8). Although it is well-established that telomeres shorten with aging, the reasons, causes and mechanisms of this process are still unclear (53-55). The extracellular environment plays a role in the regulation of telomere length and telomerase activity, but increased oxidative stress or reduction of antioxidants can also lead to accelerated telomere shortening (56).

Telomere shortening is a strong marker of replicative capacity. Cells that reach a critical number of telomeric repetitions exit the cell cycle (57). Therefore, the study of the determinants and modifiers of telomere dynamics organization can provide valuable information regarding the telomere biology implicated in health, disease and aging. This may help identify which molecules and pathways can effectively be used as targets for treatment of deficiencies and cancer, improving the quality of life and health status of the elderly (8).

The initial telomerase activity PCR assays used a specific oligonucleotide (called CX) that overlapped several base pairs with the TS primer. The interaction between the two primers resulted in the dimerization of the primers, which generated nonspecific amplification products and affected the melting curve (7). Initially, the TRAP method employed polyacrylamide gel (PAAG) electrophoresis, either a radioactively labeled primer or the primer was incorporated into the DNA during the reaction; the readout was qualitative only (58). TRAP PCR-based assay development permitted an increase in the number of studies detecting and measuring telomerase activity (59). These studies allowed for the characterization of telomerase activity and the influence of reaction components as well as conditions for it (31). The qPCR assay measures the reaction product at the exponential phase when the amounts of reaction compounds are not a limitation (37). Moreover, the TS primer is comprises a non-telomeric sequence, which makes specific amplification possible through complementarity during PCR cycles. The reverse primer (ACX) allows the products of extension to be directly related to telomerase activity (31).

This study showed that the TRAP qPCR assay is time efficient and can differentiate telomerase activity between unstimulated and stimulated cells, evidencing telomeres reactivation upon cell activation. This kind of information can be applied to different lines of research, such as aging, cancer, and chronic disease. Furthermore, we were also able to perform the assay without using the T4 protein, used in many studies, which represents one of the main advantages of our protocol, *i.e.*, reduced costs and the number of steps of the assay (33,37,60). In addition, our assay is also highly cost-effective, with an estimated cost of US$ 2 per test, which is definitely cheaper than the available commercial kits.

## CONCLUSIONS

As telomeres are important parameters, especially in aging and development of chronic diseases, a method to evaluate telomerase activity that is cost-effective and can be executed in economically compromised countries, is important to expand studies that would provide more insights into the process of aging and potentially indicate therapeutic targets to improve elderly quality of life.

In summary, we established an in-house, cost-effective and reproducible TRAP qPCR assay to measure telomerase activity. Assessment of telomerase activity is an important tool in the investigation of cellular senescence. It can contribute to the design of better approaches for the management of chronic diseases and cancer associated with the accelerated aging phenotype. Thus, an approach that is accessible and applicable under different conditions is valuable for future researchers.

## AUTHOR CONTRIBUTIONS

Pinto TNC performed the qPCR assays as well as analyzed and interpreted the data. Fernandes JR performed cell culture, and analyzed and interpreted the data. Arruda LB provided technical advice on the experimental design and reviewed the manuscript. Duarte AJS provided all the structure and equipment. Benard G was a major contributor in correcting the manuscript. All authors read and approved the final version of the manuscript.

## Figures and Tables

**Table 1:** Protein and DNA Quantity in Unstimulated and PHA-stimulated PBMCs after Culture Measured by NanoDrop.

Unstimulated DNA (ng/μL)	Stimulated DNA (ng//μL)	Unstimulated Protein (mg/mL)	Stimulated Protein (mg/mL)**
78.1	160.7	0.910	1.850
24.3	323.2	0.130	0.240
-	-	1.140	1.360
40	41.6	0.470	0.520
53.1	105.7	1.670	2.720
49.9	47.1	0.580	0.500
68.4	120.1	1.140	2.480
93.8	118.6	1.340	1.890
71.4	232.3	2.480	5.410
59.4	73.2	1.730	1.900
48.8	54.7	0.890	0.920
70.8	116.7	1.140	2.170
17	37.9	0.170	0.350
53.3	151.3	1.390	3.090
34.3	41.6	0.380	0.440
50.9	84.6	0.580	1.110
-	-	0.760	0.790
77	106	0.840	1.070
80.6	215.7	1.290	4.000
106.5	234.3	2.410	5.240
23.5	46.7	0.150	0.520
95.2	488.6	2.740	6.160
22.4	112.3	0.740	2.180
61.4	149	2.200	4.180
76	111.3	2.510	3.430
-	-	1.630	1.680
79.9	282	0.870	3.340

**Note:** The values are absolute numbers obtained from the equipment. The values in the PHA-stimulated condition increased when compared to the unstimulated condition for DNA (*p*=0.0002) and protein (*p*<0.0001).
